# Bilateral internal iliac artery ligation in trauma patients with severe pelvic hemorrhage: A systematic review

**DOI:** 10.1371/journal.pone.0303476

**Published:** 2025-02-06

**Authors:** Soon Tak Jeong, Do Wan Kim, Wu Seong Kang

**Affiliations:** 1 Department of Physical Medicine and Rehabilitation, Ansanhyo Hospital, Ansan City, Republic of Korea; 2 Department of Thoracic and Cardiovascular Surgery, Chonnam National University Hospital, Chonnam National University Medical School, Gwangju, Republic of Korea; 3 Department of Trauma Surgery, Jeju Regional Trauma Center, Cheju Halla General Hospital, Jeju, Republic of Korea; National Trauma Research Institute, AUSTRALIA

## Abstract

**Introduction:**

Severe pelvic hemorrhage significantly contributes to mortality in trauma patients, yet the most effective treatment for severe pelvic injuries remains unclear. This systematic review evaluates the mortality and morbidity associated with bilateral internal iliac artery ligation (BIIAL) in patients experiencing severe hemorrhage from traumatic pelvic fractures.

**Methods:**

Comprehensive searches were conducted in MEDLINE PubMed, EMBASE, and Cochrane databases until February 7, 2024, to identify relevant articles. The risk of bias in observational studies was assessed using the ROBINS-I tool, which evaluates bias risk in nonrandomized intervention studies. The primary outcome was mortality following BIIAL, with the secondary outcome being complications related to the procedure.

**Results:**

The review included eight studies, all observational. The overall mortality rate after BIIAL ranged from 45.0% to 76.9%. Ischemic complications from BIIAL were infrequent. A high and unclear risk of bias due to confounding and participant selection was noted across the studies. Four studies highlighted distinct indications for BIIAL compared to angioembolization. BIIAL was employed for patients with severe hemodynamic instability or when angiography was not available.

**Conclusion:**

Due to geographical limitations and significant heterogeneity among the studies reviewed, the true effect size of BIIAL remains indeterminate. Nevertheless, further prospective studies with robust designs are necessary. BIIAL holds potential as a viable option when angioembolization is not accessible or in cases of critical patient instability.

## Introduction

Severe pelvic hemorrhage represents a major cause of death in trauma patients, often stemming from pelvic fractures and associated vascular injuries. The mortality rate associated with hemodynamically unstable pelvic injuries can reach up to 40% [1, 2]. The complex vascular network within the pelvic area, characterized by its abundant collateral circulation, complicates the management of these hemorrhages. This complexity necessitates a multidisciplinary approach and a comprehensive understanding of the anatomical and physiological facets of the injury.

Recently, various modalities such as pelvic binders, external pelvic fixation, preperitoneal pelvic packing (PPP), angioembolization, and resuscitative endovascular balloon occlusion of the aorta (REBOA) have been introduced [3–5]. Current guidelines for controlling severe pelvic hemorrhage include these procedures [3–5]. However, the optimal modality and the most effective combination remain unclear. A recent retrospective cohort study utilizing the American College of Surgeons Trauma Quality Improvement Program database found that only pelvic angioembolization was associated with a reduction in mortality compared to PPP and zone 3 REBOA [6]. Conversely, a network meta-analysis showed a favorable outcome of external fixation over PPP and angioembolization [7]. In cases of hemodynamically unstable pelvic hemorrhage, various factors and settings may serve as confounders, complicating the identification of a clear causal relationship. The availability of various procedures varies significantly across institutions and countries, influenced by differences in medical resources or the skill set and knowledge of medical practitioners.

The internal iliac artery, also known as the hypogastric artery, is the primary source of pelvic hemorrhage following traumatic pelvic fractures. It supplies blood to the pelvic viscera, pelvic wall, genitalia, buttocks, and medial side of the thigh [8]. During angioembolization, swift embolization of the bilateral internal iliac arteries has been employed for damage control in cases of severe pelvic hemorrhage [9]. Similarly, bilateral internal iliac artery ligation (BIIAL) has been used in various medical contexts [8]. However, the effectiveness of BIIAL in managing traumatic pelvic hemorrhage remains uncertain. Recent guidelines on hemodynamically unstable pelvic fractures have not included the use of BIIAL [3–5].

This systematic review aims to investigate the mortality and morbidity associated with BIIAL in patients experiencing severe hemorrhage due to traumatic pelvic fractures.

## Methods

### Published study search and selection criteria

This study was conducted in accordance with the Preferred Reporting Items for Systematic Reviews and Meta-Analyses guidelines [10] (S1 Checklist). The study protocol was registered prospectively in PROSPERO (CRD42023457867; https://www.crd.york.ac.uk/prospero). We identified relevant articles through exhaustive searches of the MEDLINE PubMed, EMBASE, and Cochrane databases up to February 7, 2024. These databases were queried using the keywords: (((internal iliac artery) OR (hypogastric artery)) AND ligation) AND (trauma* OR injur*). An additional search involved a thorough review of the reference lists of relevant articles. We screened all retrieved articles by their titles and abstracts to determine their suitability for inclusion. Review articles and meta-analyses were assessed to identify further studies meeting the eligibility criteria. Subsequently, the search results were carefully evaluated, selecting studies that focused on bilateral internal iliac artery ligation in patients with pelvic injuries and associated hemorrhage for inclusion.

The primary outcome was mortality following bilateral internal iliac artery ligation. The secondary outcome was complications related to bilateral internal iliac artery ligation. The inclusion criteria for this review were: (i) trauma patients diagnosed with traumatic pelvic injury and concurrent hemorrhage; (ii) patients who underwent bilateral internal iliac artery ligation as a treatment for pelvic hemorrhage; (iii) comparisons between bilateral internal iliac artery ligation and angioembolization; (iv) availability of relevant outcomes, such as mortality or morbidity, in the reported data; and (v) provision of odds ratios (ORs), means with standard deviations, or data that allowed their calculation. Studies lacking sufficient information, focusing on diseases other than the specified conditions, nonoriginal research articles like case reports, and publications not in English were excluded from the analysis.

### Data extraction

Two investigators performed data extraction on all studies deemed eligible. They gathered essential information from each study, including the first author’s name, publication year, study location, design, duration, the total number of patients analyzed, patient age, injury severity score (ISS), vital signs, the specific surgical technique employed for bilateral internal iliac artery ligation, any additional surgical interventions such as preperitoneal pelvic packing or angioembolization, complications arising from the bilateral internal iliac artery ligation, and the mortality rate. We defined complications from bilateral internal iliac artery ligation as limb ischemia, rebleeding in the pelvis, pelvic infection, ischemia, and damage to nearby organs or vessels.

### Quality assessment

For the evaluation of bias risk in observational studies, we employed the ROBINS-I tool, which has been previously used for assessing bias risk in nonrandomized intervention studies [11]. Two independent investigators conducted the review of all studies. Any differences in the selection of studies and the extraction of data were resolved through consensus.

## Results

### Selection and characteristics

**Fig 1:**
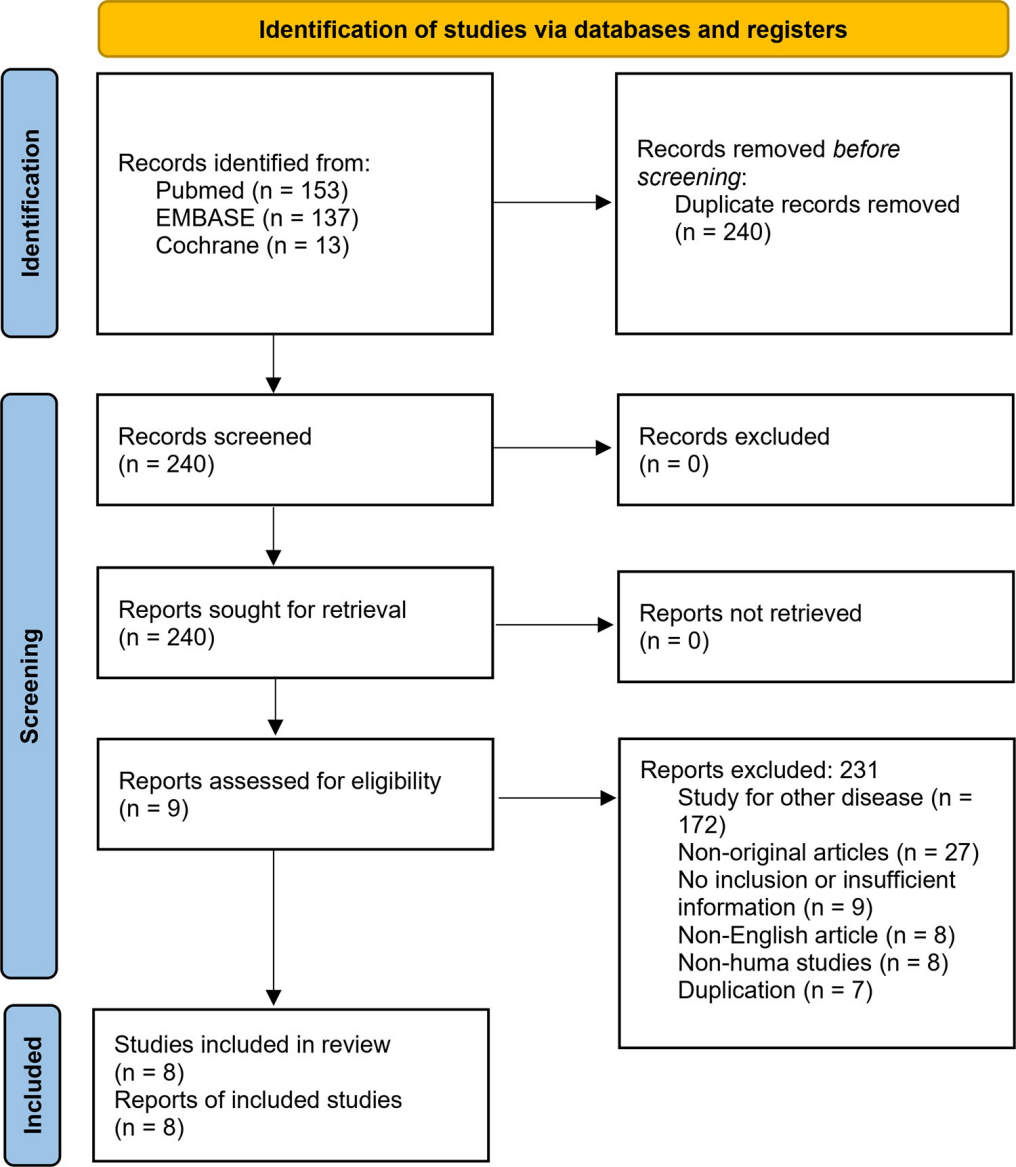
Flowchart summarizing literature and study selection.

A systematic search identified 240 studies. Among these, 232 were deemed ineligible and subsequently excluded from the analysis for the following reasons: 172 studies were focused on diseases not related to the topic of interest, 27 were not original research, 9 lacked the required inclusion criteria or did not provide adequate information, 8 were published in languages other than English, and 7 were duplicates. Ultimately, eight studies [12–19] satisfied the predetermined eligibility criteria and were included in this systematic review (Fig 1).

### Included and excluded studies

Table 1 summarizes detailed information on the studies that met the eligibility criteria. All the studies were observational, with none being randomized. Three studies conducted at the same single center in the US [12, 15, 18] suggest the possibility of patient duplication across these studies. Similarly, three studies from a single center in China [12, 13, 16] may also have overlapping patients. One study was a multicenter study [17], whereas the remaining [12–16, 18, 19] were conducted in single centers. One study was excluded for lacking sufficient information upon full-text review [20].

**Table 1:** Summary of eligible studies.

Author	Year	Location	Study period	Study design	Indication of BIIAL	Patient	Intervention	Comparison	Outcome
Gao et al. [12]	2005	China, single center	1990–2003	Retrospective	ND	79 patients with pelvic fracture with high suspicion for injury of adjacent viscera, 41 patients with massive bleeding	BIIAL (33/41), angioembolization (8/41), PEF (not reported number of patients)	None	7/79 (8.9%) overall mortality, one right common iliac artery thrombosis
Yang et al. [13]	2008	China, single center	1995–2005	Retrospective	ND	41 patients with severe multiple trauma	BIIAL (21/29), angioembolization (2/29), PEF (10/29), damage control (+)	None	12.2% mortality (5/41), including BIIAL and angioembolization
DuBose et al. [14]	2010	USA, single center	2006–2008	Retrospective	Hemodynamically unstable patients unresponse to resuscitation and packing, precluded transport to angiography	28 patients with pelvic fracture with acute development of hemodynamic instability unresponsive to aggressive fluid and blood product administration	BIIAL (28/33, 20 suture ligations, 1 Rummel tourniquet, 7 clip occlusions) and PPP (21/33) or external fixation (7/33), damage control (+). ED thoracotomy (1/33)	Survivor vs. nonsurvivor, excluding death due to severe brain injury	18/28 (64%) mortality, 10/21 (47.6%) mortality after excluding seven due to severe brain injury, no ischemic complications due to BIIAL
Chernobylsky et al. [15]	2018	USA, single center	2004–2017	Retrospective	Surgeon’s discretion,	163 pelvic hemorrhage patients	BIIAL (51/163, silastic loop ligation, no permanent ligation), angioembolization (112/163), damage control (+)	BIIAL vs. angioembolization	Decreased mortality in angioembolization (23% vs. 57%, *p* < 0.01).
					hemorrhagic shock with evidence of unstable pelvic fracture (49%, most common indication), decompensated before arrival of interventional radiology (in either ED or CT scanner)				
									fewer blood product in angioembolization (PRBC, FFP, platelets).
									fewer infections in angioembolization (5.7% vs. 14%, *p* = 0.07).
									delayed pelvic bleeding (4.5% in angioembolization vs. 22% in BIIAL. *p* = 0.004)
Huang et al. [16]	2019	China	2006–2015	Retrospective	ND	68 patients with multiple injuries, severe pelvic fractures and hemodynamic instability having received surgical hemostasis	BIIAL (49/68), angioembolization (14/68), both BIIAL and angioembolization (5/68), PEF (53/68), pelvic packing (2/68), damage control (+)	BIIAL vs. angioembolization	19/68 (27.9%) overall mortality including other BIIAL and angioembolization
Jang et al. [17]	2022	South Korea, three trauma centers	2015–2018	Retrospective	ND	157 patients with pelvic fracture who had hemodynamic instability	BIIAL (13/157), REBOA (27/157), PPP (89/157), angioembolization (66/157), PEF (20/157), damage control (+)	Survivor vs. nonsurvivor	10/13 (76.9%) mortality in BIIAL;
									8/13 (61.5%) mortality due to acute hemorrhage in BIIAL
Schellenberg et al. [18]	2022	USA, single center	2008–2020	Retrospective	Surgeon’s discretion	77 patients with blunt traumatic pelvic hemorrhage	Temporary BIIAL using vessel loop (77/77), damage control (+),ED thoracotomy (33/77)	None	54/77 (70.1%) mortality in BIIAL;
									no local complications related to BIIAL
Choi et al. [19]	2023	South Korea, single center	2017–2021	Retrospective	Hemodynamically unstable patients who do not respond to initial fluid resuscitation	20 patients with severe pelvic trauma with hemodynamic instability	BIIAL (20/20, ligation by silk) with PPP (ligation after PPP), damage control (+)	None	9/20 (45.0%) overall mortality, 2/20 (10%) pelvic rebleeding, 1/20 (5%) lower extremity artery occlusion, 2/20 (15.0%) iliac vein injury

BIIAL, bilateral internal iliac artery ligation; ND, not descripted; PPP, preperitoneal pelvic packing; ED, emergency department; CT, computed tomography; PRBC, packed red blood cells; FFP, fresh frozen plasma; ED, emergency department

### Quality assessment

**Fig 2:**
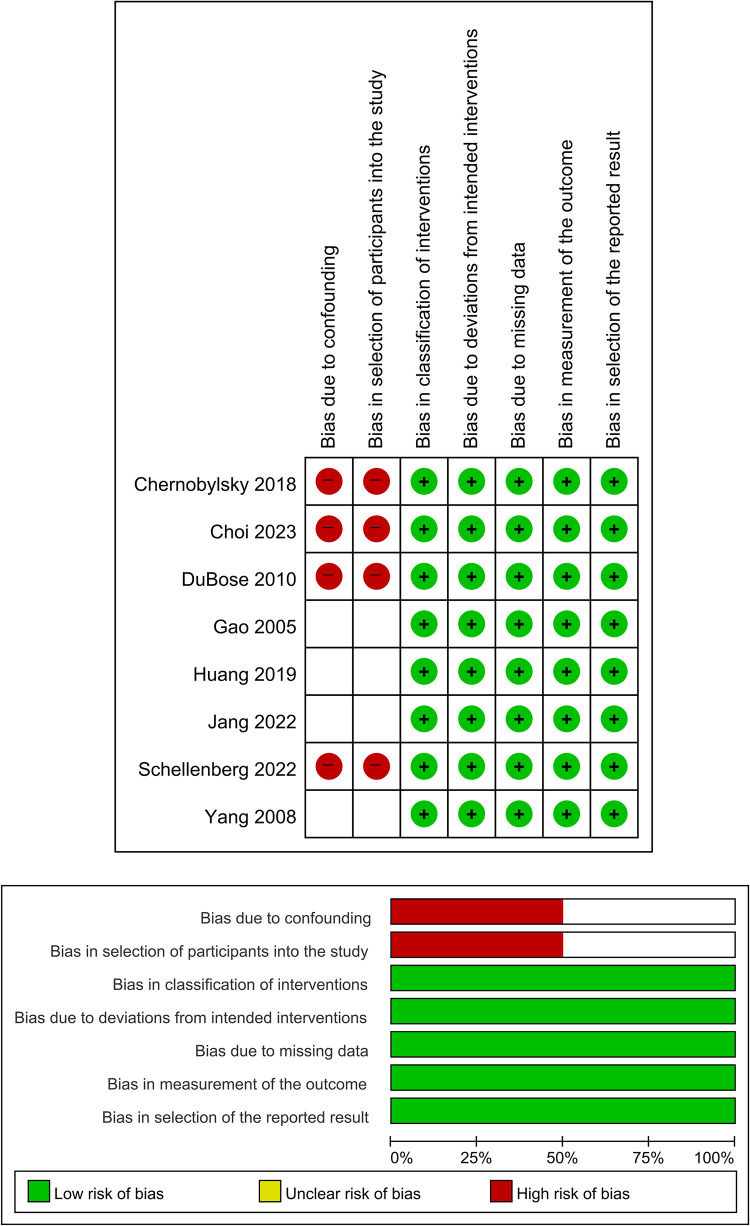
(A) Risk of bias graph: Review authors’ assessments of each risk of bias item, presented as percentages across all included studies. (B) Risk of bias summary: Review authors’ assessments of each risk of bias item for each included study.

Overall, all studies under consideration were retrospective and observational. We determined that four studies [14, 15, 18, 19] exhibited a high risk of bias concerning confounding and selection of participants, as indicated by their application of different criteria for BIIAL compared with angioembolization. In contrast, the other four studies [12, 13, 16, 17] did not provide clear criteria for BIIAL, leading us to classify these as having an unclear risk of bias (Fig 2). None of the studies were assessed as having a low risk of bias in terms of confounding and selection of participants.

### Detailed procedure of BIIAL

With respect to the procedural details, three studies outlined that BIIAL was executed by carefully dissecting distally along the common iliac artery in the retroperitoneal space to locate the internal iliac artery. [14, 18, 19] Meanwhile, four studies failed to provide a detailed description of the procedure. [12, 13, 16, 17] In three studies [14, 15, 18], a temporary BIIAL was performed using a silastic vessel loop and clip during the initial damage control laparotomy, which was removed in a subsequent operation. On the other hand, Dubose et al. [14] achieved a 70.1% rate of permanent occlusion (20/28), and Choi et al. [19] achieved a 100% rate of permanent ligation using silk suture ties (20/20).

### Indication of BIIAL

In three studies from a US trauma center [14, 15, 18], the indication for BIIAL was at the surgeon’s discretion and was performed in patients who were not suitable for transfer to the angiographic suite because of hemodynamic instability. Conversely, stable patients were subjected to angiography. Choi et al. carried out BIIAL in hemodynamically unstable patients who did not respond to fluid resuscitation, while stable patients were evaluated using computed tomography and angiography as well [19]. Four studies did not specify the criteria for BIIAL [12, 13, 16, 17].

### Other procedures for controlling pelvic hemorrhage

Seven studies descripted damage control surgery incorporating BIIAL [13–19]. In China, three studies documented the use of both BIIAL and angioembolization among their subjects [12, 13, 16]. Notably, these studies observed a higher incidence of BIIAL compared to angioembolization [12, 13, 16]. Pelvic external fixation [12–14, 17], PPP [14, 17, 19], and REBOA [17] were also documented. A single study highlighted the routine application of PPP alongside BIIAL [19].

### Mortality following BIIAL

The mortality rates post-BIIAL, as reported in five studies, ranged between 45.0% and 76.9%. [14, 15, 17–19] Chernobylsky et al. observed a lower mortality rate in cases of angioembolization compared to BIIAL (23% vs. 57%, *p* < 0.01) [15]. Dubose et al. recorded a 47.6% mortality rate after excluding patients who died due to severe brain injuries [14]. Jang et al. attributed a 61.5% mortality rate to acute hemorrhage following BIIAL [17]. Meanwhile, three studies that included both angioembolization and BIIAL reported mortality rates of 8.9%–27.9% [12, 13, 16]. These studies, however, did not specify mortality exclusively associated with BIIAL.

### Complications related BIIAL

Gao et al. identified a case of right common iliac artery thrombosis, while Choi et al. noted an instance of lower extremity artery occlusion [12, 19]. Additionally, Choi et al. described iliac vein injuries during surgery in two patients [19]. Dubose et al., however, reported no ischemic complications attributable to BIIAL [14]. Only one study compared the outcomes of BIIAL with angioembolization, revealing lower rates of infection (5.7% vs 14%, *p* = 0.07) and delayed bleeding (4.5% vs 22%, *p* = 0.004) in the angioembolization group [15]. Furthermore, Choi et al. documented two instances of pelvic rebleeding following BIIAL [19]. Remarkably, Schellenberg et al. found no local complications associated with BIIAL, underscoring that ischemic complications post-BIIAL were infrequent [18].

## Discussion

Our systematic review reports on the outcomes of BIIAL in treating traumatic pelvic hemorrhage. Although several eligible studies have highlighted a significantly elevated mortality rate associated with BIIAL, the patient phenotypes significantly diverged from those treated with angioembolization. Patients who were more severe and unstable received BIIAL as opposed to angioembolization. Notably, in a certain study [19], BIIAL was employed when angioembolization was unavailable due to a lack of medical resources. In one specific country, the frequency of BIIAL procedures for pelvic hemorrhage surpassed that of angioembolization [12, 13, 16]. Further prospective research is needed to establish the precise indications and the extent of its effectiveness. To our knowledge, this is the first systematic review focused on BIIAL for the treatment of traumatic pelvic hemorrhage. BIIAL could play a crucial role in the “damage control” strategy for managing unstable pelvic injuries.

The management of pelvic bleeding poses a considerable challenge to trauma surgeons due to the rich blood supply and the complex anatomy of the vascular system, which complicates the effective control of hemorrhage. Recent guidelines on the management of hemodynamically unstable pelvic hemorrhage have introduced a multimodal treatment approach, including angioembolization, PPP, pelvic binders, external fixation, and REBOA [3–5]. However, BIIAL has yet to be incorporated into these guidelines [3–5]. Our review indicates that BIIAL has been applied in a restricted number of regions and for a limited set of indications. Currently, BIIAL is not regarded as a widely applicable treatment modality compared to other procedures such as angioembolization or PPP [3–5]. Alternatives to BIIAL for severe pelvic hemorrhage were summarized in Table 2 [4, 9].

**Table 2:** Alternatives to bilateral internal iliac artery ligation for severe pelvic hemorrhage.

Treatment modality	Pros	Cons
Angioembolization	Useful for controlling arterial hemorrhage.	Does not directly control venous or bony bleeding.
	Minimal invasive.	Requires a specialist with proficient endovascular skills, who may not be available 24/7.
	Available for both diagnosis and treatment.	Repeated angiography may be required due to rebleeding in some cases.
	Nonselective embolization can be performed for damage control.	Selective angiography may require a longer procedure time.
Preperitoneal pelvic packing	Can be performed within 20 minutes by experienced surgeons	Does not directly control arterial bleeding
	Easy to learn	
	Provides a tamponade effect to control venous or bony bleeding	
		Additional angiography may be required if bleeding continues
	Advantageous when angiography is unavailable or would result in significant delay	
External pelvic fixation	Provides a tamponade effect, decreasing pelvic volume to prevent further bleeding	Does not directly control arterial bleeding
		Further angiography may be required in many cases.

Angioembolization, involving selective or nonselective embolization of pelvic arteries, is considered a highly effective hemostatic technique for managing pelvic hemorrhage [9]. This approach offers numerous benefits, including reduced morbidity and the ability to target specific sources of bleeding. Analogous to BIIAL, proximal embolization of bilateral internal iliac arteries is recognized as a critical “damage control” strategy for patients suffering from exsanguination due to pelvic injuries [9, 21]. A previous systematic review and meta-analysis demonstrated that proximal embolization of the internal iliac artery resulted in positive outcomes with a minimal morbidity rate [9]. Our systematic review identified a study indicating superior outcomes of angioembolization over BIIAL. However, angioembolization requires the availability of a well-trained specialist with an endovascular skill set and access to specialized equipment, such as an angiographic suite. Importantly, a comprehensive medical team comprising interventionists, nurses, and paramedics must be prepared to respond to emergencies, including unstable patients experiencing exsanguination. In our review, Choi et al. noted the lack of a 24-hour availability of interventional radiologists, necessitating the use of BIIAL in place of angioembolization [19]. Indeed, three studies conducted in China observed a higher frequency of BIIAL procedures compared to angioembolization [12, 13, 16]. Of note, recent investigations have documented the performance of angioembolization by trauma surgeons [22, 23]. Nonetheless, its application remains confined to a limited number of trauma centers. Consequently, BIIAL could serve as a viable alternative to angioembolization under certain conditions.

The concept of “damage control” has significantly influenced trauma surgery by enhancing hemorrhagic control and reducing mortality rates [24, 25]. PPP has been introduced as an effective damage control procedure for managing severe pelvic hemorrhage [26]. Utilizing PPP enables rapid control of hemorrhage through a tamponade effect on venous or bony source bleeding, while decreasing the volume of the retroperitoneal space. Nevertheless, PPP does not directly control arterial bleeding, and the tamponade effect may be compromised if the peritoneum is opened during surgery. Recent systematic reviews and meta-analyses have shown that over a quarter of PPP cases require additional angioembolization for effective hemorrhage management [27]. Although PPP proves beneficial when angioembolization is not immediately available, subsequent angiography might be necessary [27]. In contrast, BIIAL appears to offer a more effective and potent hemostatic procedure than PPP, despite its increased invasiveness. Contemporary treatment paradigms consider various approaches, including the use of a pelvic binder, PPP, angiography, or REBOA, as complementary rather than competing strategies [3–5, 28]. Yet, BIIAL has not been integrated into these existing treatment methodologies although one guideline for battlefield treatment descripted the BIIAL [29]. Our systematic review may offer novel insights into the management of severe pelvic hemorrhage for trauma surgeons.

Surgical ligation or embolization of the internal iliac artery has been reported across various specialties [8]. It has been found that the reasons for ligation or embolization of the internal iliac artery include obstetrics and gynecology in 25.1% of cases, vascular surgery in 25.1%, oncology in 17.5%, and trauma in 4.1% [8]. Our systematic review found limited reports on the surgical ligation of both internal iliac arteries in trauma cases. BIIAL involves identifying the internal iliac artery and proceeding with its ligation, either permanently or temporarily. The artery can be accessed through a trans-abdominal or retroperitoneal approach, with the trans-abdominal approach offering the advantage of identifying both iliac arteries within the same surgical field and providing a beneficial perspective for identifying other intra-abdominal injuries. However, this approach carries the potential risk of injuring adjacent organs, such as the iliac vein, internal iliac vein, or ureter. In our review, Choi et al. reported an iliac vein injury during the BIIAL procedure [19]. A primary concern with BIIAL is the potential for ischemic sequelae, such as pelvic ischemia. Our systematic review noted a small number of ischemic complications. A recent meta-analysis on proximal embolization of internal iliac arteries indicated a lower incidence of ischemic complications [9]. The use of gelfoam for embolization, thought to temporarily obstruct arterial flow, resulted in fewer ischemic complications. Interestingly, studies in the US reported temporary ligation with a silastic vessel loop in our systematic review [14, 15, 18]. However, these studies did not discuss long-term outcomes, such as the vascular patency of the internal iliac artery post-temporary ligation. Nevertheless, temporary ligation of the internal iliac artery may serve as a valuable strategy to mitigate ischemic sequelae. Further investigation is necessary, given that this practice has been reported in the US [14, 15, 18].

Our study presents several limitations. Firstly, it included only observational studies, as no randomized controlled trials were identified through our search. Prospective studies may pose challenges for unstable patients with pelvic injuries. Secondly, we were unable to calculate a pooled effect size because of insufficient information and heterogeneity among the studies considered. Additionally, patient duplication might have occurred as some studies were conducted in the same hospital, which precluded the performance of a meta-analysis. Thirdly, our review was limited to articles written in English. Lastly, there was a scarcity of data regarding long-term outcomes following BIIAL.

## Conclusion

The true effect size of BIIAL on patient outcomes remains uncertain due to geographical limitations and significant heterogeneity in the studies reviewed. This systematic review encountered challenges related to data adequacy and study diversity, which obstructed the possibility of conducting a meta-analysis. Nevertheless, further prospective studies with robust designs are imperative to ascertain the actual impact of BIIAL. Despite these limitations, BIIAL could serve as a viable option in the absence of angioembolization or under conditions of severe patient instability, offering a potential alternative to angiography in resource-constrained settings.

## Supporting information

S1 Checklist PRISMA 2020 checklist. (PDF)

S2 Checklist Human subjects research checklist. (DOCX)

## Abbreviations

PPP: preperitoneal pelvic packing
REBOA: resuscitative endovascular balloon occlusion of the aorta
BIIAL: bilateral internal iliac artery ligation
ORs: odds ratios
ISS: injury severity score
